# Venous Blood Gas (VBG) Analysis Is as Safe and Equally Reliable as Arterial Blood Gas (ABG) Analysis in the Determination of Prognosis in Chronic Liver Disease Patients: A Study Conducted in a Tertiary Care Hospital in Uttar Pradesh, India

**DOI:** 10.7759/cureus.86977

**Published:** 2025-06-29

**Authors:** Manish K Bansal, Veenavadinee Mishra, Raghav Singhal, Mayank Sharma, Chandra Prakash, Sapna Rawat

**Affiliations:** 1 Internal Medicine, Sarojini Naidu Medical College, Agra, IND; 2 General Medicine, Sarojini Naidu Medical College, Agra, IND; 3 Gastroenterology, Sarojini Naidu Medical College, Agra, IND; 4 Critical Care Medicine, Sarojini Naidu Medical College, Agra, IND; 5 Critical Care Medicine, Sanjay Gandhi Postgraduate Institute of Medical Sciences, Lucknow, IND; 6 Emergency Medicine, Sarojini Naidu Medical College, Agra, IND; 7 General Internal Medicine, Sarojini Naidu Medical College, Agra, IND

**Keywords:** acid–base disturbance, arterial blood gas (abg), child–turcotte–pugh (ctp) score, chronic liver disease (cld), hepatic encephalopathy, hospital mortality, lactate levels, non-invasive diagnostics, prognostic markers, venous blood gas (vbg)

## Abstract

Introduction

Chronic liver disease (CLD) frequently causes systemic complications, including acid-base disturbances, significantly influencing patient prognosis. Arterial blood gas (ABG) analysis is traditionally utilized to monitor these disturbances, but presents procedural risks, especially in patients with coagulopathies, which is a well-known complication of chronic liver disease (CLD). Venous blood gas (VBG) analysis has emerged as a safer alternative, yet its prognostic significance in CLD requires further investigation.

Aim & objective

This study aimed to evaluate the prognostic significance of venous blood gas parameters in chronic liver disease patients. The objectives included assessing VBG parameters in CLD patients, correlating these parameters with disease severity using the Child-Turcotte-Pugh (CTP) score, and determining their predictive role in patient outcomes and mortality.

Methodology

A hospital-based observational cross-sectional study was conducted from November 2022 to January 2025 at S. N. Medical College, Agra, including 253 patients diagnosed with CLD. VBG parameters such as pH, pCO₂, bicarbonate (HCO₃⁻), base excess, and serum lactate were measured upon admission. Clinical severity was classified using the CTP score, and outcomes including complications, hospital stay, and mortality were analyzed. Data analysis employed descriptive and inferential statistical methods using SPSS version 26 (IBM Corp., Armonk, NY, USA).

Results

Among participants, 63.24% were male, with alcohol-related liver disease being the most common etiology (47.8%). Significant differences were observed in VBG parameters across CTP classes, with lower pH (7.21±0.09) and bicarbonate (16.3±4.1 mmol/L) and elevated lactate (4.9±1.7 mmol/L) in CTP Class C (p<0.001). Strong correlations existed between lactate levels and CTP scores (r=0.82, p<0.001). Elevated lactate (>4.5 mmol/L) was the most potent independent mortality predictor (adjusted HR=7.1). Kaplan-Meier analysis showed significantly decreased 30-day survival rates with increased lactate and worsening CTP class (p<0.001).

Conclusion

VBG parameters, particularly elevated lactate and decreased pH, provide critical prognostic information in CLD patients, correlating strongly with disease severity and mortality. Incorporating VBG analysis in routine clinical practice enhances risk stratification and management of hospitalized CLD patients, offering a safer, reliable, and practical alternative to arterial blood gas analysis.

## Introduction

Arterial blood gases (ABG) testing is commonly used to determine oxygenation and acid-base status, but is associated with rare but serious complications such as infections, bleeding, nerve injury, vascular injury, and limb loss. Multiple studies have looked at the venous blood gas (VBG) as a less invasive alternative for routine monitoring of acid-base status, especially in the coagulopathy scenario like chronic liver disease [1, 2]. Portal and splanchnic complications to chronic liver disease, along with complications relating to the systemic and pulmonary circulation, affect the prognosis of the patient as part of a multi-organ syndrome. Splanchnic vasodilatation in relation to portal hypertension is responsible for the hyperdynamic circulation and abnormal distribution of blood volume. Due to splanchnic pooling of blood, there is a reduced “effective arterial blood volume” and activation of baroreceptor and volume-receptor reflexes as the outcome. The enhanced vasodilatation and counterregulatory overactivity of vasoconstrictor systems play major roles in the development of the multi-organ failure in cirrhosis with impaired function and perfusion of kidneys, lungs, brain, skin, and muscles. Blood gas analysis is considered essential to reach the final diagnosis, decide the adequate treatment, and monitor its effectiveness in the management of the patients. It is beneficial to study the hepatic role in the maintenance of acid-base homeostasis and also the effects of acute or chronic hepatic disease on the acid-base disturbances. In clinical application and practice, physicians managing patients with acute or chronic hepatic cell failure should suspect the possibility of the existence of lung-kidney-liver cross-talk. Accurate interpretation of acid-base balance is not possible without thorough assessment and detection of respiratory gas exchange. The interrelationship between pulmonary and hepatic disease is, actually, well known. Ventilation perfusion mismatching, alveolar capillary oxygen disequilibrium, or intrapulmonary or extrapulmonary shunting, or a combination of all of these, is found [3]. As chronic liver diseases and cirrhosis progress gradually, blood oxygenation may also decline over time. The occurrence of ABG alterations and hypoxemia in patients with chronic liver disease significantly influences clinical decision-making and management strategies [4].

## Materials and methods

A hospital-based observational, cross-sectional study was conducted in the Department of General Medicine at Sarojini Naidu Medical College, a tertiary care hospital in Agra (Uttar Pradesh), India, from March 2023 to January 2025. A total of 253 patients over 18 years of age who have chronic liver disease have been enrolled after obtaining informed consent. The risk, benefit, and purpose of the study were explained to all of them in their language. Participants were given the opportunity to ask questions and withdraw from the study at any time.

Ethical approval was received from the institutional ethics committee (SNMC/IEC/DHR/2025/103) of Sarojini Naidu Medical College. Confidentiality of the patients was maintained. Patients having evidence of sepsis, respiratory disease, or chronic kidney disease or those with a denial for consent were excluded from the study.

Patients were assessed through detailed history, examination, and investigations like liver function tests, coagulation profiles, viral markers (hepatitis B and C, HIV), and imaging when necessary.

Venous blood samples were collected using a heparinized 1 ml syringe under all aseptic precautions. Analysis was done using the ABL 800 FLEX blood gas analyzer and a specific calibration solution immediately after sample collection. The VBG (venous blood gases) parameters included pH, partial pressure of carbon dioxide (pCO2), partial pressure of oxygen (pO2), bicarbonates (HCO3-), base excess, and serum lactates.

Disease severity was evaluated with the Child-Turcotte-Pugh scoring system, classifying the patients into CTP Class A, B, and C. Outcomes, including complications, hospital stay, and mortality, were analyzed.

Data were entered in Microsoft Excel and analyzed using SPSS software version 26 (IBM Corp., Armonk, NY, USA). Descriptive statistics (mean, SD, frequency, and percentage) were used for baseline characteristics. Correlation analysis (Pearson/Spearman) assessed relationships between VBG values and CTP scores. Comparative analysis (t-test, ANOVA, Mann-Whitney U test) compared VBG values across CTP classes. Multivariate logistic regression identified independent predictors of poor outcome and mortality. A p-value <0.05 was considered statistically significant (at 95% confidence interval).

Table 1 and Table 2 show the distribution of study subjects according to demography and etiology, respectively.

**Table 1:** Distribution of sociodemographic profile among the study participants (N = 253) BG Prasad: Bohm Gram Prasad scale

Variable	Frequency (n)	Percentage (%)
Gender		
Male	160	63.25
Female	93	36.75
Education		
Primary	103	41.67
Illiterate	87	34.87
Secondary	63	25.00
Socioeconomic Status (BG Prasad)		
Lower Middle	81	32.40
Middle	73	29.20
Lower	44	17.60
Upper Middle	42	16.80
Upper	13	5.20

**Table 2:** Distribution of etiology of chronic liver disease among the study participants (N = 253) NAFLD: Nonalcoholic fatty liver disease

Etiology	Frequency (n)	Percentage (%)
Alcohol-related	121	47.8%
Hepatitis B	63	24.9%
Hepatitis C	34	13.4%
NAFLD	22	8.7%
Others (autoimmune, genetic, drug induced, etc.)	13	5.1%

Out of the total study participants (N=253), 36.75% were female (93) and 63.25% were male (160). In terms of education, 41.67% of participants had primary education (103), 34.87% were illiterate (87), and 25% had secondary education (63). Regarding socioeconomic status, 32.40% of participants belonged to the lower middle class (81), 29.20% to the middle class (73), 17.60% to the lower class (44), 16.80% to the upper middle class (42), and 5.20% to the upper class (13).

Out of the total study participants (N=253), 47.8% had alcohol-related chronic liver disease, 24.9% had hepatitis B, 13.4% had hepatitis C, 8.7% had nonalcoholic fatty liver disease (NAFLD), and 5.1% had other etiologies like autoimmune, genetic causes, etc.

## Results

**Figure 1:**
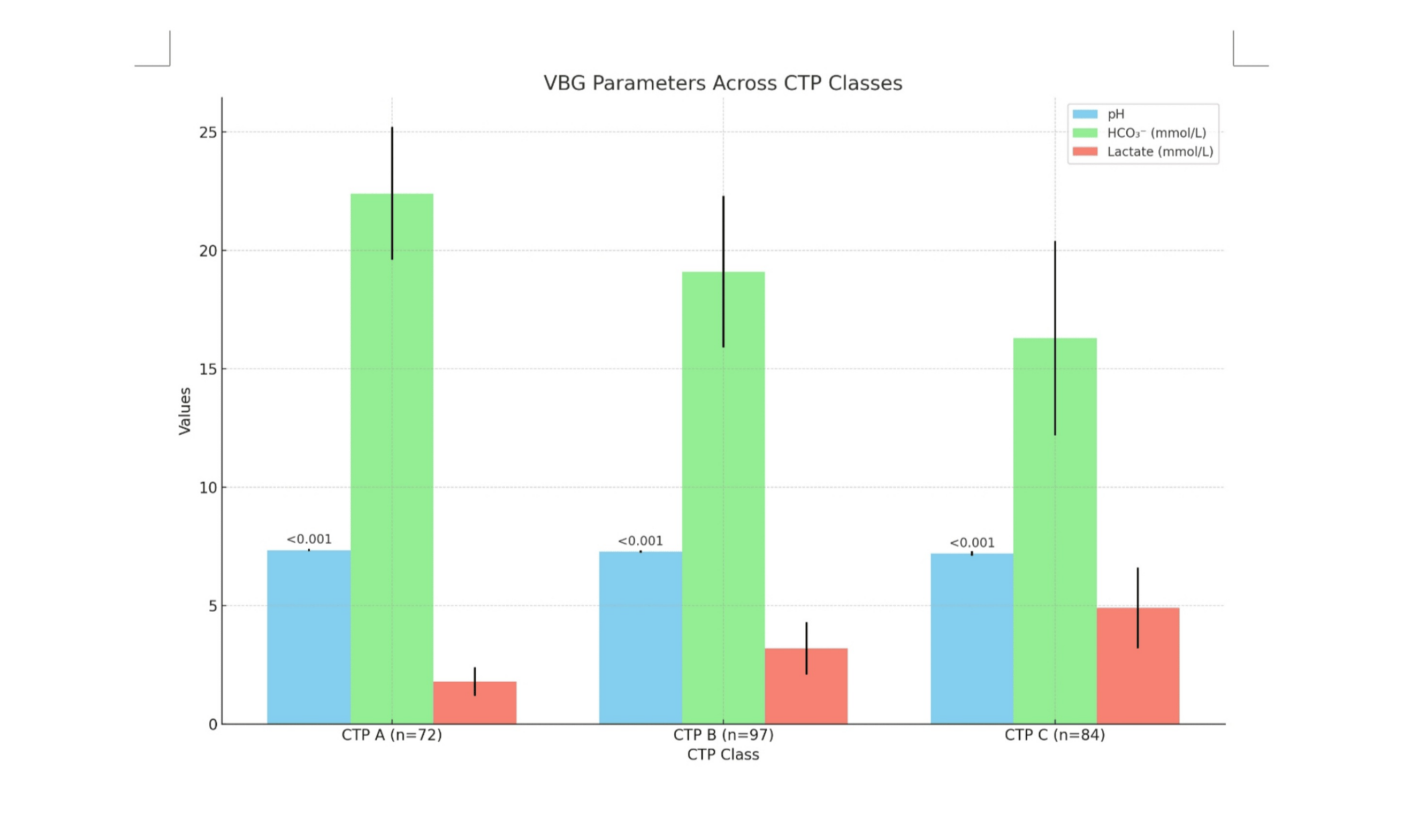
VBG Parameters Across CTP Classes (N= 253) VBG: Venous blood gases; CTP: Child-Turcotte-Pugh score

Table 3 and Figure 1 illustrate the VBG parameters across the various CTP classes, showing that out of the total study participants, 72 were classified as CTP A, 97 as CTP B, and 84 as CTP C. The pH levels were significantly different across the groups, with CTP A having a pH of 7.35 ± 0.04, CTP B 7.28 ± 0.06, and CTP C 7.21 ± 0.09, with a p-value of <0.001. HCO₃⁻ levels were highest in CTP A at 22.4 ± 2.8 mmol/L, followed by 19.1 ± 3.2 mmol/L in CTP B, and lowest in CTP C with 16.3 ± 4.1 mmol/L. Lactate levels also increased across the groups, from 1.8 ± 0.6 mmol/L in CTP A to 3.2 ± 1.1 mmol/L in CTP B and 4.9 ± 1.7 mmol/L in CTP C.

**Table 3:** VBG parameters across CTP classes (N = 253) VBG: Venous blood gases; CTP: Child-Turcotte-Pugh score

VBG Parameter	CTP A (n=72)	CTP B (n=97)	CTP C (n=84)	p-value
pH	7.35 ± 0.04	7.28 ± 0.06	7.21 ± 0.09	<0.001
HCO₃⁻ (mmol/L)	22.4 ± 2.8	19.1 ± 3.2	16.3 ± 4.1	
Lactate (mmol/L)	1.8 ± 0.6	3.2 ± 1.1	4.9 ± 1.7	

Table 4 shows the correlation between VBG parameters and the CTP score. pH had a Pearson’s r value of 0.76 with a p-value of <0.001, indicating a strong positive correlation. HCO₃⁻ and lactate also demonstrated positive correlations with the CTP score, with Pearson’s r values of 0.76, 0.71, and 0.82, respectively.

**Table 4:** Correlation between VBG parameters and CTP score VBG: Venous blood gases; CTP: Child-Turcotte-Pugh score

Parameter	Pearson’s R	P-value
pH	- 0.76	<0.001
HCO₃⁻	0.71	
Lactate	0.82	

Table 5 shows VBG parameters in patients with vs. without hepatic encephalopathy. Among the study participants, 89 had hepatic encephalopathy (HE) and 164 did not. The pH level in patients with HE was 7.24 ± 0.08, while in those without HE, it was 7.33 ± 0.05, with a significant difference (p < 0.001). The lactate level in patients with HE was 4.3 ± 1.5 mmol/L, compared to 2.1 ± 0.9 mmol/L in patients without HE, also showing a significant difference (p < 0.001).

**Table 5:** VBG Parameters in Patients With vs. Without Hepatic Encephalopathy Among the study participants VBG: Venous blood gases; HE: Hepatic encephalopathy

Parameter	With HE (n=89)	Without HE (n=164)	P-value
pH	7.24 ± 0.08	7.33 ± 0.05	<0.001
Lactate (mmol/L)	4.3 ± 1.5	2.1 ± 0.9	<0.001

Table 6 shows the impact of ascites on VBG parameters among the study participants. Out of the total study participants, 92 had no ascites, 77 had mild ascites, and 84 had moderate/severe ascites. It shows that participants with no ascites had a pH of 7.34 ± 0.05 and lactate levels of 2.0 ± 0.8, while those with mild ascites had a pH of 7.29 ± 0.06 and lactate levels of 3.1 ± 1.0. Participants with moderate/severe ascites had a pH of 7.20 ± 0.08 and lactate levels of 4.6 ± 1.5. The p-value indicates a significant difference in the parameters across the groups.

**Table 6:** Impact of ascites on VBG parameters among the study participants VBG: Venous blood gases

Ascites Severity	pH	Lactate (mmol/L)	p-value
None (n=92)	7.34 ± 0.05	2.0 ± 0.8	<0.001
Mild (n=77)	7.29 ± 0.06	3.1 ± 1.0	<0.001
Moderate/Severe (n=84)	7.20 ± 0.08	4.6 ± 1.5	<0.001

Table 7 shows the VBG parameters as predictors of mortality (logistic regression). Among the study participants, a pH level below 7.25 was linked to a 4.8 times higher chance of death (95% CI: 2.6-8.9, p < 0.001), a lactate level above 4.5 mmol/L was linked to a 6.2 times higher chance of death (95% CI: 3.4-11.3, p < 0.001), and a bicarbonate level below 18 mmol/L was linked to a 3.1 times higher chance of death (95% CI: 1.7-5.6, p = 0.002), all of which were important indicators of mortality.

**Table 7:** VBG parameters as predictors of mortality (logistic regression) among the study participants VBG: Venous blood gases

Parameter	Odds Ratio	95% CI	p-value
pH <7.25	4.8	2.6–8.9	<0.001
Lactate >4.5 mmol/L	6.2	3.4–11.3	<0.001
HCO₃⁻ <18 mmol/L	3.1	1.7–5.6	0.002

Table 8 shows the prognostic performance of VBG parameters (ROC analysis). Out of the total study participants, the lactate parameter demonstrated the highest prognostic performance with an AUC of 0.89 (95% CI: 0.84-0.94), a sensitivity of 82.1%, and a specificity of 88.2%. The cutoff value for lactate was >4.5, and the p-value was less than 0.001. The pH parameter followed with an AUC of 0.85 (95% CI: 0.79-0.91), a sensitivity of 75.5%, and specificity of 83.3%, with a cutoff value of <7.25 and a p-value of less than 0.001. HCO₃⁻ showed an AUC of 0.78 (95% CI: 0.71-0.85), with a sensitivity of 69.4%, specificity of 74.5%, and a cutoff value of <18, with a p-value also less than 0.001. Lastly, the pCO₂ parameter had the lowest AUC at 0.67 (95% CI: 0.59-0.75), with a sensitivity of 58.2%, specificity of 65.1%, and a cutoff value of >45, with a p-value of 0.002.

**Table 8:** Prognostic performance of VBG parameters (ROC analysis) VBG: Venous blood gases; ROC: Receiver-operating characteristic curve

Parameter	AUC (95% CI)	Cutoff Value	Sensitivity	Specificity	p-value
Lactate (mmol/L)	0.89 (0.84–0.94)	>4.5	82.1%	88.2%	<0.001
pH	0.85 (0.79–0.91)	<7.25	75.5%	83.3%	<0.001
HCO₃⁻ (mmol/L)	0.78 (0.71–0.85)	<18	69.4%	74.5%	<0.001
pCO₂ (mmHg)	0.67 (0.59–0.75)	>45	58.2%	65.1%	0.002

## Discussion

Regarding the venous blood gas (VBG) parameters across CTP classes, our study revealed that the pH decreased progressively from CTP A (7.35 ± 0.04) to CTP B (7.28 ± 0.06) and CTP C (7.21 ± 0.09), with a highly significant p-value (<0.001). HCO₃⁻ declined from 22.4 ± 2.8 mmol/L (CTP A) to 19.1 ± 3.2 mmol/L (CTP B) and further to 16.3 ± 4.1 mmol/L (CTP C). Similarly, lactate levels increased from 1.8 ± 0.6 mmol/L in CTP A to 3.2 ± 1.1 mmol/L in CTP B and 4.9 ± 1.7 mmol/L in CTP C. However, no significant correlation was observed for pCO₂ levels in ABG due to chronic lung disease (CLD), suggesting that pCO₂ may not be a reliable indicator for assessing CLD in these patients [5-7]. In terms of correlation between VBG parameters and CTP score, our findings revealed a strong correlation with pH showing r = 0.76 (p < 0.001), pCO₂ r = 0.68, HCO₃ r = 0.71, and lactate r = 0.82 [8].

In the present study, the venous blood gas (VBG) parameters between patients with and without hepatic encephalopathy showed marked differences. Patients with HE had a significantly lower pH (7.24 ± 0.08) and higher lactate levels (4.3 ± 1.5 mmol/L) compared to non-HE patients, who had a pH of 7.33 ± 0.05 and lactate of 2.1 ± 0.9 mmol/L with p-values <0.001 [5]. The present study showed that as ascites severity increased, pH declined and lactate rose [8]. The present study highlights the logistic regression where pH <7.25 (OR = 4.8), lactate >4.5 mmol/L (OR = 6.2), and HCO₃ < 18 mmol/L (OR = 3.1) were significant predictors of mortality, all with p <0.01 [8].

In the present study, comparison of VBG in survivors vs. non-survivors: Among the study participants in our study, the mean pH value in survivors was 7.31 ± 0.06, while in non-survivors, it was significantly lower at 7.18 ± 0.09 (p < 0.001) [8]. Lactate levels in survivors were 2.4 ± 1.1 mmol/L, whereas non-survivors showed elevated levels of 4.8 ± 1.6 mmol/L (p < 0.001) [7].

Length of hospital stay by lactate levels among the study participants in our study: participants with lactate levels <2.5 mmol/L (n=112) had an average hospital stay of 6.2 ± 2.1 days; those with lactate between 2.5-4.5 mmol/L (n=95) stayed for 9.5 ± 3.3 days; and individuals with lactate >4.5 mmol/L (n=46) stayed the longest at 14.8 ± 5.6 days. The p-value was <0.001, indicating strong statistical significance [6, 8].

The present study illustrates ROC analysis where lactate had the highest AUC of 0.89, sensitivity of 82.1%, specificity of 88.2%, and a cutoff of >4.5. pH had AUC of 0.85, sensitivity of 75.5%, and specificity of 83.3% with a cutoff <7.25. HCO3 and pCO2 had lower prognostic value [8]. The present study reveals that in multivariate analysis, CTP class C had an adjusted HR of 5.3 (p<0.001), lactate >4.5 mmol/L had HR 7.1 (p<0.001), pH <7.25 had HR 3.9 (p=0.001), and lower socioeconomic status had HR 2.5 (p=0.008). These values reflect significant independent associations with mortality [8].

This study highlights the prognostic value of venous blood gas (VBG) analysis in chronic liver disease (CLD) but has several limitations. Its cross-sectional design limits causal inference, and the lack of long-term follow-up may underestimate outcomes. Reliance on the static Child-Turcotte-Pugh (CTP) score and exclusion of arterial blood gas (ABG) analysis or metabolic markers may reduce accuracy. The single-center setting and exclusion of patients with common comorbidities limit generalizability. Technical variability in VBG measurements and a modest sample size further constrain findings. Future multicenter, longitudinal studies with broader inclusion and ABG comparison are recommended for validation.

## Conclusions

Based on the findings of this study, venous blood gas (VBG) analysis should be integrated into the routine assessment of patients with chronic liver disease (CLD), particularly those classified as Child-Turcotte-Pugh (CTP) B or C, given its strong prognostic value. Parameters such as pH, lactate, and HCO₃⁻ demonstrated significant correlations with disease severity and mortality, with lactate >4.5 mmol/L and pH <7.25 emerging as critical thresholds for poor outcomes. However, the value of venous blood pCO2 was not found to be significant in this study, showing its shortcoming with respect to ABG (arterial blood gases). These markers should be used for early risk stratification, enabling timely interventions in high-risk patients, such as those with hepatic encephalopathy (HE) or severe ascites, where metabolic acidosis and hyperlactatemia were pronounced. Overall, the study highlights the utility of VBG as a simple, cost-effective tool and equally useful as ABG for prognostication and clinical decision-making in chronic liver disease, with the potential to enhance patient outcomes through early risk identification and tailored management strategies.
